# Impact of vaccination on COVID-19 severity during the second wave in Brunei Darussalam, 2021

**DOI:** 10.5365/wpsar.2024.15.1.992

**Published:** 2024-03-25

**Authors:** Chee Fui Chong, Muhd Syafiq Abdullah, Pui Lin Chong, Rosmonaliza Asli, Babu Ivan Mani, Natalie Raimiza Momin, Justin Wong, Noor Affizan Rahman, Jackson Tan, Vui Heng Chong

**Affiliations:** a National Isolation Centre, Ministry of Health, Tutong, Brunei Darussalam.

## Abstract

**Objective:**

Coronavirus disease (COVID-19) vaccinations have been shown to prevent infection with efficacies ranging from 50% to 95%. This study assesses the impact of vaccination on the clinical severity of COVID-19 during the second wave in Brunei Darussalam in 2021, which was due to the Delta variant.

**Methods:**

Patients included in this study were randomly selected from those who were admitted with COVID-19 to the National Isolation Centre between 7 August and 6 October 2021. Cases were categorized as asymptomatic, mild (symptomatic without pneumonia), moderate (pneumonia), severe (needing supplemental oxygen therapy) or critical (needing mechanical ventilation) but for statistical analysis purposes were dichotomized into asymptomatic/mild or moderate/severe/critical cases. Univariate and multivariable analyses were conducted to identify risk factors associated with moderate/severe/critical disease. Propensity score-matched analysis was also performed to evaluate the impact of vaccination on disease severity.

**Results:**

The study cohort of 788 cases (mean age: 42.1 ± 14.6 years; 400 males) comprised 471 (59.8%) asymptomatic/mild and 317 (40.2%) moderate/severe/critical cases. Multivariable logistic regression analysis showed older age group (≥ 45 years), diabetes mellitus, overweight/obesity and vaccination status to be associated with increased severity of disease. In propensity score-matched analysis, the relative risk of developing moderate/severe/critical COVID-19 for fully vaccinated (two doses) and partially vaccinated (one dose) cases was 0.33 (95% confidence interval [CI]: 0.16–0.69) and 0.62 (95% CI: 0.46–0.82), respectively, compared with a control group of non-vaccinated cases. The corresponding relative risk reduction (RRR) values were 66.5% and 38.4%, respectively. Vaccination was also protective against moderate/severe/critical disease in a subgroup of overweight/obese patients (RRR: 37.2%, *P* = 0.007).

**Discussion:**

Among those who contracted COVID-19, older age, having diabetes, being overweight/obese and being unvaccinated were significant risk factors for moderate/severe/critical disease. Vaccination, even partial, was protective against moderate/severe/critical disease.

By February 2023, the total number of reported cases of coronavirus disease (COVID-19) exceeded 757 million and over 6.8 million lives had been lost globally. (*1*) The rapid development and approval for emergency use of multiple novel COVID-19 vaccines within a year of detection of the first cases represented a pivotal moment in the global effort to reduce the impact of the pandemic. (*2*-*6*) The first emergency use authorization of a COVID-19 vaccine was made by the United States Food and Drug Administration on 11 December 2020, after the completion of phase 3 trials that demonstrated efficacy in preventing symptomatic infection with severe acute respiratory syndrome coronavirus 2 (SARS-CoV-2) of up to 95%. (*2*, *7*) Vaccination programmes commenced shortly afterwards, and several COVID-19 vaccines were prequalified for emergency use by the World Health Organization (WHO).

During 2021, several studies confirmed that vaccination was highly effective in reducing symptomatic SARS-CoV-2 infection, as well as the risk of hospitalization, serious illness and death. (*8*) However, cases of breakthrough infection after vaccination were reported, especially after the emergence of the Delta strain of SARS-CoV-2 (B.1.617.2). For all three prequalified mRNA vaccines, efficacy against infection with the Delta variant dropped to below 80%. (*9*) Neutralization with post-vaccination sera assay studies also showed a 19- to 42-fold reduction in neutralizing activity against the Beta variant (B.1.351). (*10*) However, vaccination remained important for reducing the risk of infection and severe disease and mitigating the impact of COVID-19.

In Brunei Darussalam, the first wave of COVID-19 started on 9 March 2020 and was rapidly controlled, with the last community spread documented on 6 May 2020. Control measures included public health and social measures such as mask wearing and restrictions on movements and social gatherings, coupled with testing, close monitoring and surveillance of cases, and regular review and updating of infection control and outbreak management protocols in response to the evolving nature of the pandemic. (*11*) An important part of the national response, and one that was instrumental in the containment of the first wave, was the establishment of a designated centre, the National Isolation Centre (NIC), to isolate and treat all positive cases. (*11*) Brunei Darussalam started rolling out vaccination with four WHO-prequalified vaccines (Vaxzevria [AstraZeneca], BBIBP-CorV [Sinopharm], Comirnaty [Pfizer-BioNTech] and Spikevax [Moderna]) on 3 April 2021, 4 months before the start of the second wave on 7 August 2021. The second wave was due to the Delta variant of SARS-CoV-2, (*11*) a more contagious variant than the original and Alpha strains.

This study assesses the effectiveness of vaccination in preventing severe disease among patients with COVID-19 in Brunei Darussalam during the second wave and investigates the role of vaccination in modifying selected known risk factors for severe to critical COVID-19.

## Methods

### Study design

This study used a retrospective cohort study design to assess the impact of vaccination on the risk of developing severe COVID-19 among patients who were admitted to the NIC during the second wave of the COVID-19 outbreak in Brunei Darussalam between 7 August and 6 October 2021.

### Setting

The management of the COVID-19 outbreak in Brunei Darussalam has been previously described. (*11*) In brief, at the start of the second wave, all patients with COVID-19 were admitted to the NIC. However, over the course of the second wave, increasing numbers of mild cases of COVID-19 were admitted to the newly established community isolation centres for isolation and treatment. Symptomatic patients with moderate or severe disease, as well as those with mild disease plus significant comorbidities (i.e. diabetes, obesity, older age and end-stage renal failure) and persistent fever, dyspnoea or diarrhoea continued to be admitted to the NIC for management and treatment.

### Study population

Patients included in the study were those admitted to the NIC between 7 August and 6 October 2021, who tested positive for COVID-19 through laboratory-confirmed reverse transcription-polymerase chain reaction (RT–PCR) testing. To counter the effect of changing NIC admission criteria and ensure equal representation across the spectrum of COVID-19 disease severity in the study population, patients were randomly selected (using Microsoft Excel’s random number generator) at three time points (early August, mid-September and early October). Patients aged ≤ 18 years, pregnant women and patients with end-stage renal disease were excluded, as these patient subgroups were not eligible for vaccination at the time of the start of the second wave.

### Data collection

Patient data were prospectively collected using a specially designed database that was set up to monitor and aid the management of patients admitted to the NIC. Information on patients’ demographic characteristics (age, sex) was collected, as well as data on relevant clinical risk factors such as body mass index (BMI), diabetes mellitus, hypertension and dyslipidaemia. Patients’ vaccination status was retrieved from patients’ Bru-HIMS health records and coded as either “complete” (if patients had received their second dose at least 14 days before contracting COVID-19), “partial” (if patients had received their first dose at least 14 days before contracting COVID-19 or less than 14 days had elapsed since their second dose) or “unvaccinated” (if patients were unvaccinated before contracting COVID-19 or less than 14 days had elapsed since their first dose). (*4*)

### Clinical severity categories

Patients were assigned to one of five categories according to clinical severity: asymptomatic, mild (symptomatic without pneumonia), moderate (clinical or radiological evidence of pneumonia), severe (moderate respiratory decompensation requiring non-invasive supplementary oxygen) and critical (respiratory decompensation requiring intubation and mechanical ventilation or extracorporeal membrane oxygenation support). (*11*) Patients’ clinical severity was recorded daily for management decision-making.

### Primary outcome

The primary outcome was defined as the highest clinical severity category attained by the patient during their hospitalization. For the purposes of subsequent statistical analyses, the outcome variable was dichotomized into two categories: asymptomatic/mild disease and moderate/severe/critical disease. Patients who died were included in the moderate/severe/critical category, irrespective of cause of death. Deaths were recorded as a COVID-19 death if supported by evidence of COVID-19 pneumonia.

### Statistical analysis

All statistical analyses were performed using IBM SPSS software (version 26). Patient characteristics, stratified by disease severity, were summarized in a descriptive analysis. Continuous data were presented as a mean ± standard deviation and compared using the independent Student’s *t*-test. Categorical data were presented as frequency and percentages and compared using Pearson’s χ^2^ test. Univariate analyses, with disease severity as the outcome, were used to explore which potential risk factors (demographic characteristics, clinical risk factors and vaccination status) were associated with more severe disease. Significant risk factors (*P* < 0.05) derived from univariate analysis were then input into a multivariable logistic regression model to calculate the odds ratio (OR) for each significant variable.

To investigate the effectiveness of vaccination in reducing the clinical severity of COVID-19 cases, patients were matched 1:1 according to their vaccination status (vaccinated or unvaccinated) using propensity scores derived from summing the probabilities of being vaccinated given patients’ demographic characteristics (age and sex) and the presence of selected clinical risk factors (diabetes, hypertension, hyperlipidaemia and overweight/obesity). These probabilities were derived using binary logistic regression. The relative risk (RR) of more severe COVID-19 disease comparing the two propensity score-matched groups (vaccinated and unvaccinated) was estimated using a 2x2 contingency table χ^2^ test. This comparison was repeated in a subgroup analysis designed to determine the effect of complete and partial vaccination status on disease severity. Additional analyses were conducted in selected COVID-19 patient subgroups: patients aged ≥ 45 years, overweight/obese patients and patients with diabetes mellitus. Estimates of the relative risk reduction (RRR), the absolute risk reduction (ARR) and the number needed to treat (NNT) were also derived from the analyses of the matched groups. *P* < 0.05 was considered statistically significant.

## Results

### Study population

**Fig. 1:**
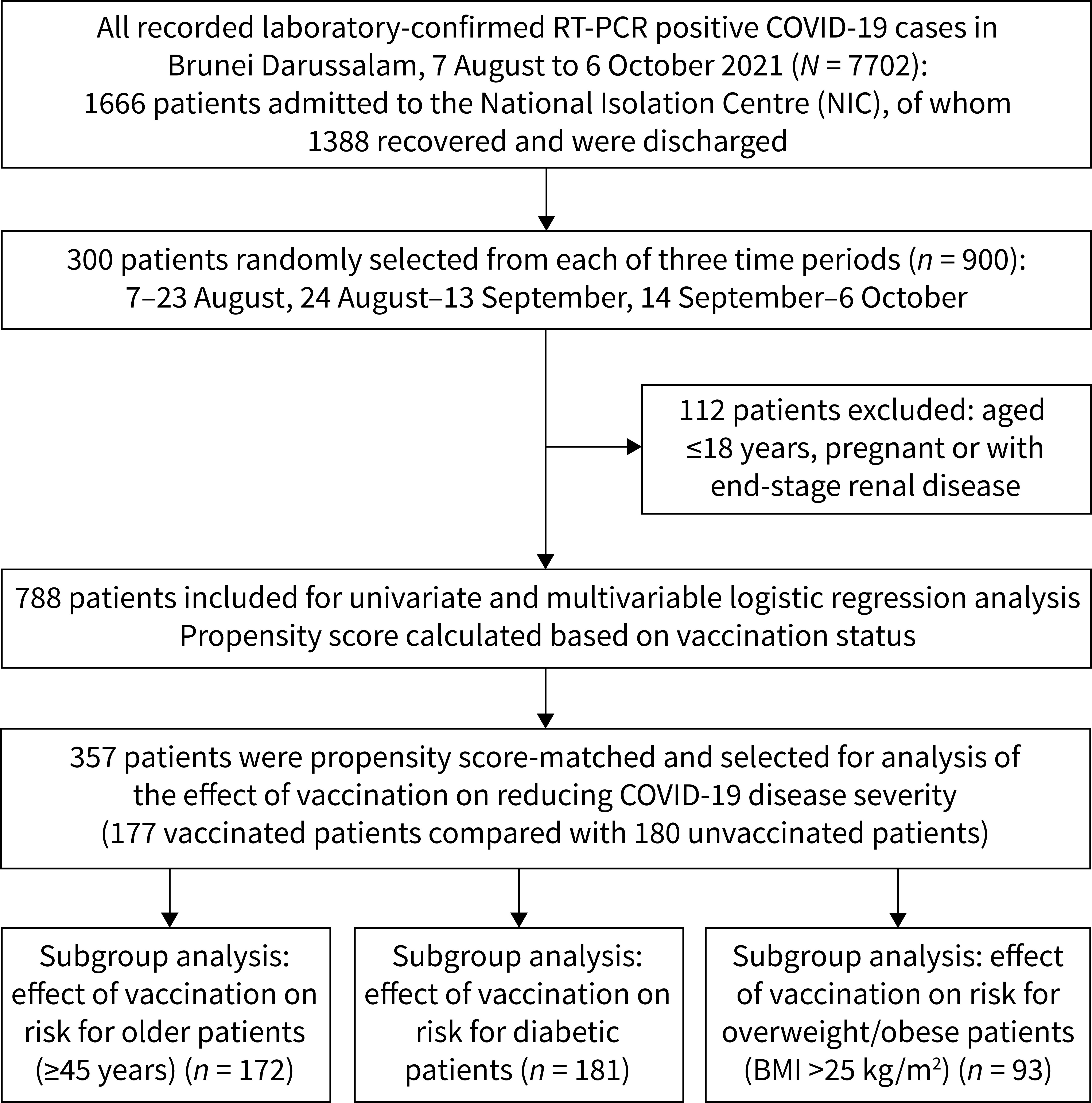
Flowchart showing recruitment of COVID-19 cases into the study, Brunei Darussalam, 7 August to 6 October 2021

Between 7 August and 6 October 2021, there were 7702 recorded cases of COVID-19 in Brunei Darussalam, of which 1666 were admitted to the NIC. Nine hundred patients were randomly selected (300 from each of three time periods), 112 of whom were subsequently excluded based on the above-mentioned exclusion criteria. The study population thus comprised a total of 788 patients (**Fig. 1**).

[**insert** Figure 1]

The mean age of patients was 42.1 ± 14.6 years; 400 were male and 388 were female (**Table 1**). Over half (*n* = 471) had either asymptomatic or mild COVID-19 (14.7% and 45.1%, respectively); of the remaining 317 patients, 169 (21.4%) were categorized as moderate cases, 127 (16.1%) as severe and 21 (2.7%) as critical. Most asymptomatic/mild patients were aged < 40 years; severe and critical patients were older, with a mean age of at least 50 years (**Table 1**).

**Table 1:** Demographic characteristics and clinical risk factors of 788 COVID-19 cases, by disease category, admitted to the National Isolation Centre between 7 August and 6 October 2021, Brunei Darussalam

Characteristic/ risk factor	*N*	Disease severity					*P* ^a^
		Asymptomatic *n* (% of total)	Mild *n* (% of total)	Moderate *n* (% of total)	Severe *n* (% of total)	Critical *n* (% of total)	
**Demographic characteristics**							
**Age (mean ± SD)**		39.13 ± 14.53	37.57 ± 13.10	44.90 ± 13.57	52.16 ± 13.94	50.05 ± 14.80	**< 0.001**^b^
**Age group**							
< 30 years	109	33 (28.5)	117 (33.0)	25 (14.8)	6 (4.7)	2 (9.5)	**< 0.001**^c^
30–39 years	130	31 (26.7)	101 (28.4)	40 (23.7)	19 (15.0)	2 (9.5)	
40–49 years	173	23 (19.8)	78 (22.0)	41 (24.2)	25 (19.7)	6 (28.6)	
50–59 years	193	14 (12.1)	31 (8.7)	40 (23.7)	40 (31.5)	5 (23.8)	
≥ 60 years	183	15 (12.9)	28 (7.9)	23 (13.6)	37 (29.1)	6 (28.6)	
**Sex**							
Male	400	61 (52.6)	175 (49.3)	87 (51.5)	64 (50.4)	13 (62.0)	0.822^c^
Female	388	55 (47.4)	180 (50.7)	82 (48.5)	63 (49.6)	8 (38.0)	
**Clinical risk factors**							
**Diabetes mellitus**							
Yes	146	12 (10.3)	37 (10.4)	43 (25.4)	48 (37.8)	6 (28.6)	**< 0.001**^c^
No	642	104 (89.7)	318 (89.6)	126 (74.6)	79 (62.2)	15 (71.4)	
**Hypertension**							
Yes	237	32 (27.6)	76 (21.4)	55 (32.5)	66 (52.0)	8 (38.1)	**< 0.001**^c^
No	551	84 (72.4)	279 (78.6)	114 (67.5)	61 (48.0)	13 (61.9)	
**Dyslipidaemia**							
Yes	158	15 (12.9)	46 (13.0)	40 (23.7)	49 (38.6)	8 (38.1)	**< 0.001**^c^
No	630	101 (87.1)	309 (87.0)	129 (76.3)	78 (61.4)	13 (61.9)	
**Overweight/obesity**							
Yes	409	49 (42.2)	165 (46.5)	105 (62.1)	75 (59.1)	15 (71.4)	**< 0.001**^c^
No	379	67 (57.8)	190 (53.5)	64 (37.9)	52 (40.9)	6 (28.6)	
**Vaccination status**							
Complete	75	19 (16.4)	38 (10.7)	11 (6.5)	7 (5.5)	0 (0)	**< 0.001**^c^
Partial	162	34 (29.3)	71 (20.0)	38 (22.5)	16 (12.6)	3 (14.3)	
Unvaccinated	551	63 (54.3)	246 (69.3)	120 (71.0)	104 (81.9)	18 (85.7)	
**Total sample**	**788**	**116**	**355**	**169**	**127**	**21**	

SD: standard deviation.

^a^ Bold *P*-values are statistically significant (< 0.05).

^b^ Analysis of variance (ANOVA) comparison of mean age.

^c^ Pearson’s χ^2^ test.

Around one third (*n* = 237, 30.1%) of patients had received at least one dose of a COVID-19 vaccine (75 completed two doses, 162 completed one dose); 551 (69.9%) were unvaccinated. Within the critical severity category (*n* = 21), there were 18 unvaccinated patients (85.7%) and three (14.3%) partially vaccinated patients. None of the critical patients had been fully vaccinated. There were 28 deaths in the study sample; in this group of patients, 22 (78.6%) were unvaccinated, 6 (21.4%) were partially vaccinated and none were fully vaccinated.

### Univariate and multivariable logistic regression analyses

Univariate analysis showed that age, diabetes mellitus, hypertension, dyslipidaemia, overweight/obesity and vaccination status were significantly associated with COVID-19 disease severity (**Table 1**).

After adjustment in a multivariable logistic regression analysis, age, diabetes mellitus, overweight/obesity and vaccination status remained significantly associated with COVID-19 disease severity. The odds of developing moderate/severe/critical disease were significantly lower in those who had been vaccinated, even partially (OR: 0.45, 95% confidence interval [CI]: 0.30–0.67, *P* < 0.001) (**Table 2**).

**Table 2:** Adjusted odds ratios for the association between selected risk factors and the risk of severe COVID-19 disease in a cohort of 788 cases admitted to the National Isolation Centre between 7 August and 6 October 2021, Brunei Darussalam

Risk factor^a^	Asymptomatic/mild *n*(%)	Moderate/severe/critical *n*(%)	*P* ^b^	Odds ratio	Lower CI	Upper CI
**Age group (1)**	133 (28.2)	176 (55.5)	** < 0.001**	2.96	2.05	4.26
**Diabetes mellitus (1)**	49 (10.4)	97 (31.0)	** < 0.001**	2.70	1.65	4.41
**Overweight/obesity (1)**	214 (45.4)	195 (61.5)	**0.001**	1.75	1.28	2.42
**Hypertension (1)**	108 (22.9)	129 (40.7)	0.213	1.36	0.84	2.18
**Dyslipidaemia (1)**	61 (13.0)	97 (30.6)	0.228	0.73	0.44	1.22
**Vaccination status**						
Complete (1)	57 (12.1)	18 (5.7)	**0.007**	0.44	0.24	0.80
Partial (2)	105 (22.3)	57 (18.0)	** < 0.001**	0.45	0.30	0.67

CI: confidence interval.

^a^ Risk factors included here are those that were statistically significant in univariate analysis: Age group (≥ 45 years = 1, < 45 years = 2), Diabetes mellitus (Yes = 1, No = 2),

Overweight/obesity (Yes = 1, No = 2), Hypertension (Yes = 1, No = 2), Dyslipidaemia (Yes = 1, No = 2) and Vaccination status (Complete = 1, Partial = 2, Unvaccinated = 3).

^b^ Bold *P*-values are statistically significant (< 0.05).

### Propensity score-matched analyses

A total of 357 patients were matched on their propensity score for vaccination: 177 vaccinated (65 complete and 112 partial) and 180 unvaccinated. There were no significant differences in the demographic and clinical characteristics between the vaccinated and unvaccinated groups, indicating that the propensity score matching produced similar comparison groups (**Table 3**). Being vaccinated (either fully or partially compared with no vaccination) decreased the risk of severe disease, with a RR of 0.62 (95% CI: 0.46–0.82), a RRR of 38.5% (95% CI: 18.9–53.8%), an ARR of 0.17 (95% CI: 0.17–0.54) and a NNT of 6 (95% CI: 2–6) (**Table 4**). Similar values were obtained when the analysis was restricted to the partially vaccinated subgroup (*n* = 111) (RR: 0.62, 95% CI: 0.46–0.82; RRR: 38.4%, 95% CI: 18.0–53.8%, respectively). However, for those who were fully vaccinated (two doses), the reduction in risk (compared with no vaccination) was higher still (RR: 0.33, 95% CI: 0.16–0.69; RRR: 66.5%, 95% CI: 31.2–83.7%, respectively) (**Table 5**).

**Table 3:** Demographic characteristics and clinical risk factors of 357 COVID-19 cases that were propensity score matched by vaccination status

Variables	Vaccinated^a^ *n* (% of total)	Unvaccinated *n* (% of total)	*P* ^b^
**Total**	177	180	
**Age (mean ± SD)**	45.85 ± 14.54	45.72 ± 14.61	0.93
**Age group**			
< 30 years	23 (48.9)	24 (51.1)	1.00
30–39 years	43 (49.4)	44 (50.6)	-
40–49 years	41 (50.0)	41 (50.0)	-
50–59 years	35 (50.0)	35 (50.0)	-
≥ 60 years	35 (49.3)	36 (50.7)	-
**Sex**			
Male	95 (49.0)	99 (51.0)	0.83
Female	82 (50.3)	81 (49.7)	-
**Clinical risk factors**			
**Diabetes mellitus**			
Yes	49 (52.7)	44 (47.3)	0.55
No	128 (48.5)	136 (51.5)	-
**Hypertension**			
Yes	80 (53.3)	70 (46.7)	0.24
No	97 (46.9)	110 (53.1)	-
**Dyslipidaemia**			
Yes	51 (52.0)	47 (48.0)	0.64
No	126 (48.6)	133 (51.4)	-
**Overweight/obesity**			
Yes	86 (47.5)	95 (52.5)	0.46
No	91 (51.7)	85 (48.3)	-

SD: standard deviation.

^a^ Vaccinated patients included those who had received at least one dose of a COVID-19 vaccine at least 14 days before their COVID-19 infection. Unvaccinated patients included those who had not received a COVID-19 vaccine or had received their first dose within 14 of their COVID-19 infection.

^b^ Pearson’s χ^2^ test used with *P* < 0.05 considered significant.

**Table 4:** Effect of vaccination status on risk of developing more severe COVID-19 in a cohort of 357 propensity score-matched cases admitted to the National Isolation Centre between 7 August and 6 October 2021, Brunei Darussalam

Vaccination status	*N*	COVID-19 severity		*P* ^b^	RR (95% CI)	RRR (95% CI)	ARR (95% CI)	NNT (95% CI)
		Asymptomatic/mild *n*(%)	Moderate/severe/ critical *n*(%)					
**Vaccinated** ^a^	177	128 (72.3)	49 (27.7)	0.001	0.62 (0.46–0.82)	38.5 (18.0–53.8)	0.17 (0.17–0.54)	6 (2–6)
**Unvaccinated**	180	99 (55.0)	81 (45.0)					

ARR: absolute risk reduction; CI: confidence interval; NNT: number needed to treat; RR: relative risk; RRR: relative risk reduction.

^a^ Vaccinated patients included those who had received at least one dose of a COVID-19 vaccine at least 14 days before their COVID-19 infection. Unvaccinated patients included those who had not received a COVID-19 vaccine or had received their first dose within 14 of their COVID-19 infection.

^b^ Pearson’s χ^2^ test used with *P* < 0.05 considered significant.

**Table 5:** Subgroup analysis to explore the effect of vaccination status (fully, partial, none) on the risk of developing more severe COVID-19 disease in a cohort of 354 propensity score-matched cases admitted to the National Isolation Centre between 7 August and 6 October 2021, Brunei Darussalam^a^

Vaccination status^a^	*N*	COVID-19 severity		*P* ^b^	RR (95% CI)	RRR (95% CI)
		Asymptomatic/mild *n*(%)	Moderate/severe/critical *n*(%)			
**Complete**	65	57 (87.7)	8 (12.3)	**0.001**	0.33 (0.16–0.69)	66.5 (31.2–83.7)
**Unvaccinated**	68	43 (63.2)	25 (36.8)			
**Partial**	111	70 (63.1)	41 (36.9)	**0.001**	0.62 (0.46–0.82)	38.4 (18.0–53.8)
**Unvaccinated**	110	44 (40.0)	66 (60.0)			

CI: confidence interval; RR: relative risk; RRR: relative risk reduction.

^a^ For subgroup analysis by vaccination status, propensity score matching resulted in the exclusion of three patients, one in the partially vaccinated and two in the unvaccinated group. Hence, the total number of patients included in this analysis was 354.

^b^ Fully vaccinated patients included those who had received two doses of COVID-19 vaccine, with the second dose administered at least 14 days before COVID-19 infection. Partially vaccinated patients were those who had received one dose of a COVID-19 at least 14 days before their COVID-19 infection; patients who had received their second dose within 14 days of their COVID-19 infection were also included in this category. Unvaccinated patients included those who had not received a COVID-19 vaccine or had received their first dose within 14 days of their COVID-19 infection.

^c^ Pearson’s χ^2^ test used with *P* < 0.05 considered significant.

Separate subgroup analyses were conducted for older patients (aged ≥ 45 years), those with diabetes mellitus and those being overweight/obese. Vaccination was significantly protective against developing more severe disease in the overweight/obese group with a RR of 0.63 (95% CI: 0.44–0.89) and a RRR of 37.2% (95% CI: 10.8–55.7%). In contrast, among the older population (aged ≥ 45 years) and those with diabetes mellitus, there was no difference in the risk of more severe disease between those who had received at least one dose of a COVID-19 vaccine and those who were unvaccinated (**Table 6**).

**Table 6:** Subgroup analysis to explore the effect of older age, overweight/obesity and diabetes on the association between vaccination status and the risk of developing more severe COVID-19 disease in a cohort of 357 propensity score-matched cases admitted to the National Isolation Centre between 7 August and 6 October 2021, Brunei Darussalam

Risk factor	COVID-19 severity		*P* ^b^	RR (95% CI)	RRR (95% CI)	ARR (95% CI)	NNT
	Asymptomatic/mild *n*(%)	Moderate/severe/critical *n*(%)					
**Older age group (≥ 45 years)**							
Vaccinated	44 (51.2)	42 (48.8)	0.17	0.81 (0.61–1.06)	19.2	0.12	9
Unvaccinated	34 (39.5)	52 (60.5)					
**Overweight/obesity**							
Vaccinated	57 (66.3)	29 (33.7)	0.007	0.63 (0.44–0.89)	37.2 (10.8–55.7)	0.20 (0.11–0.56)	5 (2–9)
Unvaccinated	44 (46.3)	51 (53.7)					
**Diabetes mellitus**							
Vaccinated	17 (34.7)	32 (65.3)	0.66	0.93 (0.70–1.23)	7.3	0.05	20
Unvaccinated	13 (29.5)	31 (70.5)					

ARR: absolute risk reduction; CI: confidence interval; NNT: number needed to treat; RR: relative risk; RRR: relative risk reduction.

^a^ Vaccinated patients included those who had received at least one dose of a COVID-19 vaccine at least 14 days before their COVID-19 infection. Unvaccinated patients included those who had not received a COVID-19 vaccine or had received their first dose within 14 of their COVID-19 infection.

^b^ Pearson’s χ^2^ test used with *P* < 0.05 considered significant.

## Discussion

Among a cohort of 788 patients who tested positive for COVID-19 and were admitted to the NIC during the second wave in Brunei Darussalam, older age, having diabetes mellitus, being overweight/obese and being unvaccinated were found to be independent risk factors for greater severity of COVID-19 disease. Our propensity score-matched analysis showed that patients who had received at least one dose of a COVID-19 vaccine were at reduced risk of developing more severe disease compared with people who were unvaccinated. In our cohort of patients, none of the fully vaccinated patients developed critical disease or died of COVID-19.

Other studies have also found that patients with these three clinical risk factors – older age, diabetes mellitus and overweight/obesity – are at increased risk for severe COVID-19 disease and death and are thus considered to be high-risk groups. (*12*-*17*) The higher risk associated with older age groups can be attributed to a waning immunity in both adaptive and innate immune responses, which has been shown to predispose older individuals to greater risk of infections and some cancers. (*18*, *19*) In a longitudinal study looking at antibody response to SARS-CoV-2 infection, 100% of patients aged 10–17 years retained their antibody titre 3 months after seropositive conversion; in those aged ≥ 40 years, this fell to 84%. (*20*) Both diabetes and obesity are on the spectrum of metabolic diseases that are associated with immune dysfunction and chronic inflammation, which increase susceptibility to COVID-19 infection. (*21*-*23*) Pulmonary function tests in obese patients, in particular those with abdominal obesity, have revealed a tendency towards restrictive respiratory patterns and reduced lung volumes compared with people with a lower BMI. (*24*) This reduction in pulmonary reserves may explain why a higher proportion of obese patients with COVID-19 decompensated rapidly and required oxygen supplementation and intubation. (*14*) Cardiovascular risk factors such as hypertension and dyslipidaemia, previously reported to be significant risk factors for severe COVID-19 infection, (*25*) were not significantly more common in the moderate/severe/critical COVID-19 group in this study. This is consistent with the findings of a study from Guangzhou, China, (*15*) and may be specific to this variant of the virus.

Our propensity score-matched analysis showed that vaccination, independently of several clinical risk factors or the type of vaccine (mRNA or inactivated), was protective against developing moderate/severe/critical COVID-19. Fully vaccinated patients, i.e. those who had received two doses of vaccine, were 67% less likely to be in the moderate/severe/critical group compared with those who were unvaccinated. Conversely, an unvaccinated patient had three times the risk of moderate/severe/critical disease than a fully vaccinated patient. Even being partially vaccinated, i.e. having received one dose of vaccine at least 14 days before infection, was associated with a RR of 0.62 and RRR of 38% (relative to unvaccinated patients).

While directly comparable studies are limited in number, our results are broadly consistent with those from other studies. For example, in a case-control study involving 119 partially vaccinated patients who were age- and sex-matched to 476 unvaccinated patients, vaccination was associated with a 69.3% RRR in death (and an ARR of 22.3%). (*26*) Likewise, a recent community-wide serosurvey conducted in 5310 subjects in Hong Kong Special Administrative Region, China, demonstrated that three or four doses of BNT162b2 or CoronaVac were effective against Omicron infection 7 days after vaccination (vaccine effectiveness ranged from 30% to 69%). (*27*) However, 100 days after vaccination, this effectiveness had waned to 6–26%. Another study involving 969 PCR-confirmed SARS-CoV-2 cases showed that 46% of the 54 fully vaccinated patients admitted to hospital developed moderate/severe/critical disease, almost twice as many as our cohort of patients with complete vaccination status. (*28*) Nevertheless, both this study and ours identified older age (≥ 80 years), overweight (BMI > 25 kg/m^2^), cardiovascular disease and diabetes as risk factors for more severe COVID-19 disease.

Our real-world findings further support and strengthen the evidence from clinical trials for the efficacy of vaccination in preventing severe COVID-19. In a study using the Comirnaty vaccine, the protective effect of the vaccine in preventing symptomatic COVID-19 infection was evident as early as 12 days after the first dose with an efficacy of 52%, which increased to 95% at 7 days after the second dose. (*3*) Similarly, a trial of Spikevax reported an efficacy of 95.2% in preventing symptomatic COVID-19 infection 14 days after the first dose. (*4*)

Based on our analysis, we estimated that the NNT to prevent one case of moderate/severe/critical disease in this cohort was six. That is to say, for every six people vaccinated with at least one dose of a COVID-19 vaccine who subsequently contracted COVID-19, one was prevented from developing moderate/severe/critical disease. This suggests that the impact of vaccination was large for the Delta variant outbreak in Brunei Darussalam. Our findings also provide strong evidence in favour of vaccination, especially in settings with limited specialist health-care resources, such as bed capacity in intensive care units, and thus limited capacity to care for severely ill COVID-19 patients. More broadly, and as repeatedly demonstrated by the COVID-19 pandemic, vaccination will undoubtedly continue to play an important role in managing disease outbreaks, even those caused by less virulent strains such as Omicron. (*29*) Preliminary analysis of more recent data collected during subsequent outbreaks of COVID-19 in Brunei Darussalam dominated by the Omicron variant, which are not reported here, suggests that the effectiveness of vaccination in protecting against moderate to critical disease remains significant despite the reduced virulence of the Omicron variant.

Our study has highlighted the fact that older patients or those with diabetes mellitus are at significantly higher risk of developing moderate/severe/critical infections, despite vaccination. (*12*, *13*) For elderly patients, breakthrough infection despite vaccination is due to immunosenescence and a reduced effectiveness of immune response to vaccination. (*19*) A recent study showed that one third of patients aged ≥ 80 years had no detectable neutralizing antibodies despite receiving two doses of a COVID-19 vaccine, whereas among those aged < 60 years, only 2.2% had no detectable neutralizing antibodies. (*30*) Data from the United States Centers for Disease Control and Prevention have confirmed that adults aged ≥ 50 years were 2–8 times more likely to be hospitalized with breakthrough COVID-19 infection despite being fully vaccinated. (*31*) It has been suggested that the same immune dysregulation and dysfunction combined with chronic inflammation may account for the increased risk seen in patients with diabetes mellitus, especially if their diabetes is suboptimally controlled. (*32*)

There are several limitations that need to be considered when interpreting our study results. First, the study group comprised a hospital-based cohort and hence the results can only be applied to other hospital settings. Second, this study did not consider the effect of treatments (e.g. steroids and remdesivir) on the outcome. However, since our primary outcome was the highest severity category obtained during hospitalization, any impact of such treatments is unlikely to have affected the allocation of the primary outcome. Third, the clinical severity categories used in our study were based on a severity scale used in South-East Asia to triage patients for admission to hospital and may differ from severity categories used elsewhere. However, our definition for the moderate/severe/critical group was equivalent to the definition of severe COVID-19 used in published randomized controlled trials evaluating the efficacy of COVID-19 vaccine candidates. (*4*) Importantly, the categories used were simple and effective for daily monitoring of patients and reporting to the Ministry of Health. Fourth, this study did not evaluate the effectiveness of the different types of COVID-19 vaccines but rather combined all vaccines (mRNA or inactivated) into a single group. In addition, this study only assessed the impact of vaccination on the Delta strain, and therefore the results will not be applicable to subsequent newer strains, such as Omicron. Lastly, we used propensity score matching to generate similar comparison groups for our analysis, thus eliminating the effect of known important confounding variables on our effect estimates for the impact of vaccination on disease severity. We consider that propensity score matching is a suitable alternative in pandemic settings where conducting a randomized control study may not be feasible, ethical or cost-effective.

In conclusion, in a cohort of patients hospitalized with COVID-19 during the second wave in Brunei Darussalam, which was dominated by the Delta variant (B.1.617.2), vaccination was effective in reducing the risk for moderate/severe/critical disease by up to 67%. For every six fully or partially vaccinated cases infected with the Delta variant, one moderate/severe/critical case can be prevented, thereby reducing health-care utilization. The protective effect of vaccination was also observed in the group of overweight or obese patients, although to a lesser degree (37%). As the pandemic progresses or transitions to an endemic phase, the severity of COVID-19 infections will continue to impact high-risk populations, and thus the case for vaccination remains.
